# Aneuploidy-driven non-heritable genomic variations demonstrate area-specific distribution in the Alzheimer’s disease brain

**DOI:** 10.1186/1750-1326-8-S1-P52

**Published:** 2013-09-13

**Authors:** Ivan lourov, Svetlana Vorsanova, Thomas Liehr, Yuri Yurov

**Affiliations:** 1 Mental Health Research Center, Russian Academy of Medical Sciences, Moscow, Russia; 2 Institute of Paediatrics and Paediatric Surgery, Ministry of Health, Moscow, Russia; 3 Department of Medical Genetics, Russian Medical Academy of Postgraduate Education, Moscow, Russia; 4 Moscow City University of Psychology and Education, Moscow, Russia; 5 Institute of Human Genetics, Jena, Germany

## Background

Post-zygotic aneuploidy is the prominent genetic feature of the human brain (1). Genomically mosaic brain results from that the excess of aneuploid neurons due to early developmental disturbances (somatic genome variations), abnormal cell cycle regulation and altered programmed cell death. As the result, aneuploidization of the brain is a likely susceptibility factor (mechanism) for brain disorders including Alzheimer’s disease.

## Materials and methods

The proportion of aneuploid cells was determined in brain areas differentially affected by neurodegeneration (prefrontal cortex, hippocampus and cerebellum) by molecular-cytogenetic and immunohistochemical techniques (interphase MFISH, immunoFISH) in brain tissues of individuals with AD and controls as described ealier (2).

## Results

Increased levels of aneuploidy (monosomy and trisomy) involving chromosome 21 and chromosome X was observed in AD brain. The high level of aneuploidy involving chromosome 21 was observed in the AD cerebrum and hippocampus. In total, the incidence of abnormal (aneuploid) neural cells was significantly higher in degenerating brain areas (hippocampus, prefrontal cortex) comparing to the less degenerating area (cerebellum).

## Conclusions

Our data indicates that AD brain areas subjected to neurodegeneration are more significantly affected by aneuploidy (especially aneuploidy of chromosomes 21 and X). We propose that widespread postzygotic aneuploidization of selected brain areas is a mechanism for AD neurodegeneration. Such area-specific distribution of aneuploidy can be explained by the accumulation of aneuploid cells during postnatal life or abnormal selective pressure against non-aneuploid cells (3). Finally, these data provide for the speculation that acquired neural aneuploidy could be generated during both developing and adult neurogenesis/gliogenesis.
